# Are orphan genes protein-coding, prediction artifacts, or non-coding RNAs?

**DOI:** 10.1186/s12859-016-1102-x

**Published:** 2016-05-31

**Authors:** Neel Prabh, Christian Rödelsperger

**Affiliations:** Department for Evolutionary Biology, Max-Planck-Institute for Developmental Biology, Spemannstrasse 35, 72076 Tübingen, Germany

**Keywords:** Orphan genes, Negative selection, Ortholog, Paralog, Nematodes, Gene expression, dN/dS

## Abstract

**Background:**

Current genome sequencing projects reveal substantial numbers of taxonomically restricted, so called orphan genes that lack homology with genes from other evolutionary lineages. However, it is not clear to what extent orphan genes are real, genomic artifacts, or represent non-coding RNAs.

**Results:**

Here, we use a simple set of assumptions to test the nature of orphan genes. First, a sequence that is transcribed is considered a real biological entity. Second, every sequence that is supported by proteome data or shows a depletion of non-synonymous substitutions is a protein-coding gene. Using genomic, transcriptomic and proteomic data for the nematode *Pristionchus pacificus*, we show that between 4129–7997 (42–81 %) of predicted orphan genes are expressed and 3818–7545 (39–76 %) of orphan genes are under negative selection. In three cases that exhibited strong evolutionary constraint but lacked expression evidence in 14 RNA-seq samples, we could experimentally validate the predicted gene structures. Comparing different data sets to infer selection on orphan gene clusters, we find that the presence of a closely related genome provides the most powerful resource to robustly identify evidence of negative selection. However, even in the absence of other genomic data, the availability of paralogous sequences was enough to show negative selection in 8–10 % of orphan genes.

**Conclusions:**

Our study shows that the great majority of previously identified orphan genes in *P. pacificus* are indeed protein-coding genes. Even though this work represents a case study on a single species, our approach can be transferred to genomic data of other non-model organisms in order to ascertain the protein-coding nature of orphan genes.

## Background

Transferring functional annotations between species constitutes an integral part of modern biomedical research that tries to prioritize candidate genes for various diseases [1, 2] as well as to better understand phenotypic evolution [3]. Although genes for which similar functions are described in one species (e.g. mouse) usually represent good candidate genes in other species of interest (e.g. human), still such strategy leads to an inherent bias in a way that only conserved genes with identifiable orthologs are pursued by functional follow up studies. This practice bears the danger of ignoring a large number of genes that either do not have identifiable one-to-one orthologs (e.g. 25 % of genes between human and mouse [4]) or completely lack any traces of homology in other species (e.g. 11 % of genes between *Caenorhabditis elegans and C. briggsae* [5]). As a consequence the latter class of taxonomically restricted genes that is as commonly referred to as orphan genes [6], new genes [7] or pioneer genes [8] has long been neglected in genetic research. Thus, it is poorly understood what role orphan genes play in the biology of their host species. However, numerous studies in vertebrates, worms and flies have shown substantial phenotypic and genomic divergence [9–12], which strongly suggests that adaptation to new biological niches is reflected by differences in gene repertoires [13]. Along these lines, our lab has recently shown that a developmental decision even if conserved between nematodes, can still be under the control of unrelated genes that have absolutely no homology between species [14]. Depending on the definition and the phylogenetic resolution, up to one third of genes in current genome sequencing projects are usually identified as orphan genes [6, 15–17]. The number of orphan genes identified in a study largely depends on the definition of orphan gene as well as on the phylogenetic resolution of available genomic data. Thus, genomes of taxonomic groups that are vastly underrepresented in genomic data predictably reveal higher number of orphan genes. Currently, we lack a comprehensive understanding of how orphan genes evolve. Strong divergence, de novo gene formation and horizontal gene transfer are frequently discussed explanations [6, 16]. However, since most genomes are at least partially annotated by gene predictions, it has been shown that orphan genes can arise from poorly assembled genomes [18]. An alternative hypothesis is that many orphan genes represent non-coding RNAs and might evolve without the constraints that are typical for protein-coding sequences. In order to investigate whether orphan genes are indeed protein-coding genes, prediction artifacts or non-coding RNAs we analysed genomic, transcriptomic and proteomic data for the nematode *Pristionchus pacificus. P. pacificus* has been established as a satellite model organism to *Caenorhabditis elegans* for comparative studies involving developmental biology, immunity [19, 20] and population genomics [21, 22]. The genome of *P. pacificus* has been sequenced using Sanger technology [23] and has been improved using 454 data [8]. Despite its relatively large size (160Mb), in terms of contiguity it is either superior or at least comparable (N50 = 1.2Mb) to many other recently published nematode genomes [16]. More importantly, it has proven of great use for several studies to identify and clone causative genes for various different phenotypes [14, 20, 24]. Various gene finding programs predicted 20,000-30,000 genes that have been evaluated several times using transcriptomic and comparative genomic data indicating that current gene annotations are largely complete [21, 23, 25].

In this study, we try to clarify the nature of orphan genes in *P. pacificus.* Our basic strategy is to first check whether they are expressed and we consider expression as positive evidence that a gene prediction is not an artifact. In addition, strong support towards the protein coding nature of a predicted gene can be gathered if it either has a match in available proteomic data or shows evidence of negative selection that manifests in depletion of nonsynomous vs. synonymous substitutions. Gene predictions that are expressed but do not fulfill any of the two criteria are candidates for non-coding RNAs. Based on the two criteria, our results show that between 40–77 % of orphan genes are actually truly protein-coding, indicating that orphan genes likely represent a substantial factor in the biology of *P. pacificus.* Application of our methodology on genomic data from other taxonomically under-sampled groups will further support the validity of orphan genes and their biological significance.

## Results

### The majority of orphan genes are transcribed

**Fig. 1:**
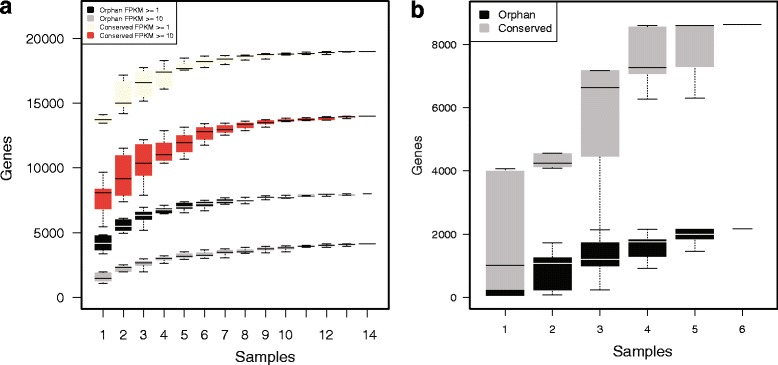
Transcription and differential expression of orphan genes. **a** RNA sequencing data from 14 experiments are used to determine the number of expressed orphan and conserved genes as a function of the number of RNA-seq samples (Y-axis) and different expression thresholds (FPKM > = 1 and FPKM > =10). The boxplot shows the variation in the number of expressed genes from ten random permutations of the order of RNA-seq samples **b**) Saturation analysis for the number of differentially expression pattern of both orphan and conserved genes from six transcriptome profiling studies [25, 28]

We used previously defined sets of 9885 orphan genes and 20,999 conserved genes [25] and 14 previously published RNA-seq experiments in *P. pacificus* to assess up to what extent both gene sets are transcribed and to examine the dependence between the amount of expressed genes and the number of included RNA-seq samples (Fig. 1a). Thereby, we defined two different thresholds on the magnitude of expression, which was measured as fragments per kilobase transcript per million fragments (FPKM) sequenced. While a value of FPKM > =10 defines robust expression, a value of FPKM > =1 can still be interpreted as reliable evidence of expression as many functionally characterized genes [26, 27] display FPKM values well below 10.

Even though conserved genes show in general higher levels of expressed (*N* = 18997,~90 %) and robustly expressed genes (*N* = 14010, ~67 %), still in our analysis 4129–7997 (42–81 %) of orphan genes are transcribed in at least one of the samples (Fig. 1a).

### Orphan genes are embedded in gene-regulatory networks

In addition to using transcription as a necessary criterion for a real gene, we also quantified the amount of conserved and orphan genes that are differentially expressed under variable conditions like different developmental stages or exposure to pathogens. The rational behind this analysis is based on the assumption that a gene that can be differentially expressed under various conditions has likely been integrated into a gene-regulatory network. Therefore such a gene is a more suitable candidate for a real gene rather than a gene (or pseudogene), which is constitutively expressed at very low levels. This would also suggest that older genes would more likely be integrated into the gene network and they will have stronger differential expression pattern compared to new genes. Consistent with this assumption, we have previously shown that orphan genes are significantly depleted among genes that are developmentally regulated and conserved genes are significantly enriched [25]. However, analysis of previously conducted transcriptome profiling studies (*N* = 6 gene sets) [28, 29] shows that not only 8623 (41 %) conserved genes but even 2165 (22 %) orphan genes are differentially expressed in *P. pacificus*. This indicates that a subset of orphan genes is differentially expressed under various conditions and implies that such an orphan gene must have persisted a sufficient amount of time in a genome for it to be integrated into a regulatory circuit.

### A subset of orphan genes shows direct evidence for translation

Next we tested to what extent conserved and orphan genes showed direct evidence of translation by testing for matches in available proteomic data [8, 30]. Using 100 % sequence identity over the full peptide length as criteria to search through the peptide data (*N* = 51,224 peptide sequences) we found peptide evidence for 428 (4 %) orphan genes. This number is quite low when compared with the 5177 (25 %) conserved genes that have peptide evidence, but it is consistent with previous study that reported [30] the under-representation of orphan genes in transcriptome and peptide data. However, we also observe that even three quarters of conserved genes do not have peptide evidence, thus indicating that the lack of peptide evidence is not a sufficient criterion to distinguish truly protein-coding genes from potential artifacts or non-coding RNAs. For this reason we employ other genomic resources to measure indirect evidence for protein-coding genes, i.e. selection against non-synonymous substitutions.

### Comparative genomics of orphan genes

**Fig. 2:**
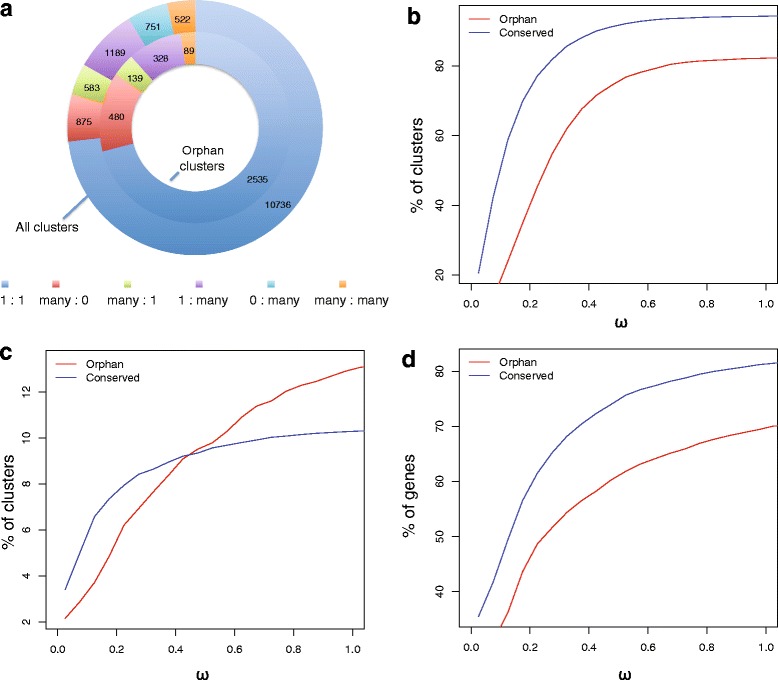
Orphan genes are under strong negative selection. **a** Distribution of gene clusters based on the number of orthologs between *P. pacificus* and *P. exspectatus*. **b** The graph shows the cumulative proportion of orphan and conserved gene clusters with evidence for negative selection relative to different ω thresholds in the *P. pacificus – P. exspectatus* orthologous clusters dataset. Using an abitrary cutoff of ω < 0.6, 78 % of all orphan clusters (*N* = 3571) show evidence of negative selection. **c** Comparison of the proportion of negatively selected orphan gene clusters and conserved gene clusters (Y-axis) based on the analysis of paralogous gene clusters. Given, that only 11 % of conserved clusters and 15 % of all orphan clusters contain *P. pacificus* paralogs, this analysis shows that even in the absence of other genomic data, negative selection can be investigated in a subset of orphan genes. **d** Cumulative proportion of orphan and conserved gene clusters under given ω value in Clade A1 – Clade A2 ortholog dataset

As most orphan genes do not display matches in peptide data, we use negative selection in protein-coding genes as an indirect evidence for translation. However, to estimate evolutionary constraint (i.e. selection against non-synonymous substitutions) at least two sequences are needed which is problematic for orphan genes since they do not have homologs in other species. This impediment can be circumvented by comparison with a species that is within the taxonomic group of the focal organism. In our case, we can make use of the recently sequenced sister species *P. exspectatus*. The genome of *P. exspectatus* shows roughly 10 % sequence divergence and was previously used to polarize intra-species comparisons within *P. pacificus* [21, 25]. To this end, all predicted genes from both species *P. pacificus* and *P. exspectatus* were segregated into 14,656 different orthologous clusters based on protein sequence homology using OrthoMCL [31]. Figure 2a shows the distribution of all the clusters into six different categories based on the number of the *P. pacificus* and *P. exspectatus* genes present in the clusters. Evidently, the majority of clusters have only two members one each from *P. pacificus* and *P. exspectatus* genomes. After filtering out *P. exspectatus* specific clusters, hybrid clusters and short peptide containing clusters or poorly aligning clusters (see *Methods*), we separated 10,327 clusters containing one or more *P. pacificus* conserved genes and 3273 clusters containing one or more *P. pacificus* orphan genes for further investigation. These two sets represent 3891 orphan and 13,103 conserved genes from *P. pacificus.* Conserved and orphan gene clusters were further subdivided into two different datasets; the first dataset called ‘orthologous clusters’ had the clusters that contain at least one *P. pacificus* and one *P. exspectatus* gene, the second dataset called ‘paralogous clusters’ consisted of all the clusters containing more than one *P. pacificus* genes minus all the *P. exspectatus* genes from the clusters. We decided to distinguish the two types of clusters in order to assess to what extent we gain evidence for negative selection with the help of a second species. The combination of both the datasets represent all *P. pacificus* genes except the singletons genes that are present only in a single copy in *P. pacificus* and do not have a corresponding homolog in the *P. exspectatus.* In order to accommodate the *P. pacificus* singletons and study selection at a closer time scale we created a third dataset called ‘clade A1-A2 orthologs’ (*N* = 30,884) where we compared divergence of orthologous gene pairs (cluster of size two) across two geographically isolated *P*_*.*_*pacificus* lineages [21].

### Orphan and conserved genes are under strong negative selection

Selection pressure was measured as the ratio of synonymous and non-synonymous rate of amino acid substitution (dN/dS) also called omega (ω) for each cluster of the three above-mentioned datasets using the PAML suit [32]. An ω = 1 would indicate neutral evolution and ω < 1 can be interpreted as evidence for negative selection. The measurement of ω on orthologous clusters showed that conserved gene clusters are under stronger negative selection than the orphan gene clusters (Fig. 2b). Nonetheless, the majority of orphan gene clusters are also under relatively strong negative selection. The result of similar analysis on paralogous conserved and orphan clusters were also comparable, keeping in mind that only 11 % of conserved clusters and 15 % of orphan clusters are present in this dataset (Fig. 2c). The selection pressure results for clade A1-A2 dataset also follows the trend of showing stronger negative selection on conserved genes when compared with orphan genes; nevertheless, the orphan genes also appear to be under robust negative selection (Fig. 2d).

### Ortholog, paralog and intra-species comparison are highly complementary

Similar to the analysis of the expression data, we define a range between a conservative and a more liberal threshold to estimate the amount of genes under negative selection. First, we arbitrarily chose ω < 0.6 as a liberal cutoff to call a cluster as evolving under negative selection. In addition, we defined a more conservative cutoff by calculating the statistical significance of a negative-selection model compared with a neutral model. For each cluster the codeml package of PAML was run twice, once allowing a single ω value for the entire cluster tree but free to change (alternate model: H_A_) and the second time ω fixed at 1 for the entire cluster tree (null model: H_0_). Likelihood ratio test was conducted with one degree of freedom and at false discovery rate (FDR) adjusted *p*-value < 0.05 for each cluster to determine the significance of the alternate model. A combination of ω < 1 and FDR adjusted *p*-value < 0.05 was used as second more conservative criterion for calling a cluster as evolving under negative selection. Please note that the lack of significance does not imply the lack of evolutionary constraint and in some cases might just be indicative of low statistical power due to the comparison of small proteins or little divergence between the sequences.

**Fig. 3:**
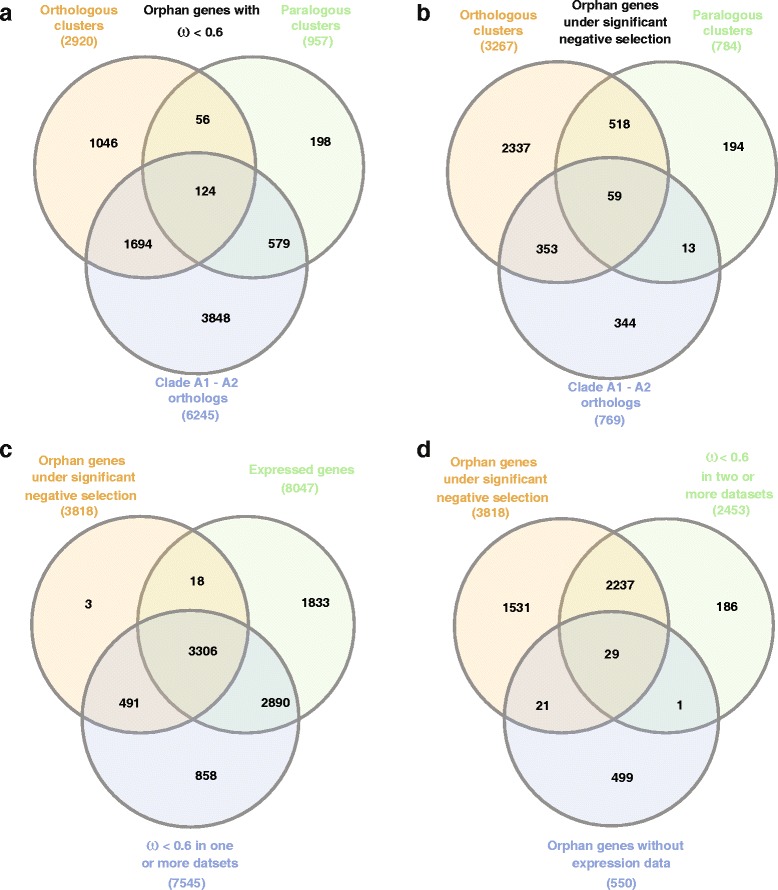
Ortholog, paralog and population genomic data are highly complementary. **a** Venn diagram for three different data sets using a definition of ω < 0.6. As most genes with evidence of negative selection are unique to one specific data set, this indicates that all three data sets are highly complementary. **b** Venn diagram for three different data sets using a definition of ω < 1 and *P* < 0.05. Again, most genes with evidence of negative selection are unique to one specific data set. **c** Overlap between different thresholds to define negative selection and expressed genes. **d** Identification of a candidate gene set of orphan genes that lack any expression data, are under significant negative selection and also have ω value less than 0.6 in at least two of the three datasets

In order to investigate the overlap between the different datasets, we decided to compare orphan candidates found to be under negative selection in all the three datasets. Taking the cutoff of ω value lower than 0.6 in any one of the three datasets (Fig. 3a) identified 7545 (76 %) of orphan genes as being under negative selection. Figure 3a shows that the largest contribution towards identified negative selection (ω < 0.6) comes from the intra-species comparison. However, since the evolutionary distances in this comparison are rather small, random fluctuations in the number of non-synonymous and synonymous sites can easily generate ω values below 0.6. Given that the signal for positive selection is unlikely to affect complete genes, ω > 1 are most likely due to such random fluctuations; based on this we estimate that around one third of the identified orphan genes with ω < 0.6 can be due to random noise. Therefore, we would like to emphasize that the cutoff of ω < 0.6 has to be regarded as upper limit of the estimated number of negatively selected orphan genes.

Using the stringent likelihood ratio test we identified 3818 (39 %) orphan genes with significant evidence for negative selection in at least one of the three data sets (Fig. 3b). More precisely, out of 3273 orthologous orphan clusters 2899 clusters (89 %) show significantly better goodness of fit for negative selection (alternate model having derived ω value less than 1) compared with the neutral evolution model (null model). The corresponding figure for the conserved clusters is 9838 out of 10,327 (95 %). Among paralogous clusters, 297 out of 514 (57 %) orphan clusters and 997 out of 1222 (82 %) conserved clusters showed significant goodness of fit for negative selection. In clade A1-A2 dataset, only 769 out of 9885 orphan gene clusters and 4767 out of 20,999 conserved gene clusters had significant goodness of fit supporting negative selection, indicating that the divergence between two *P. pacificus* lineages in general is not enough to identify robust evidence for negative selection at a single gene level.

In order to further evaluate the use of ω < 0.6 as less conservative cutoff, we compared the identified gene sets from both methods with available expression data (Fig. 3c). This demonstrates that a cutoff of ω < 0.6 on one side captures almost all examples of significant negative selection and it minimizes the fraction of genes that are neither expressed nor show significant likelihood results. We have observed that changing this cutoff value to 0.5 or 0.7 drastically impairs this balance. In summary, our analysis suggests that a large proportion (39–76 %) of orphan genes are under strong negative selection.

### Evolutionary constraint predicts expression

**Fig. 4:**
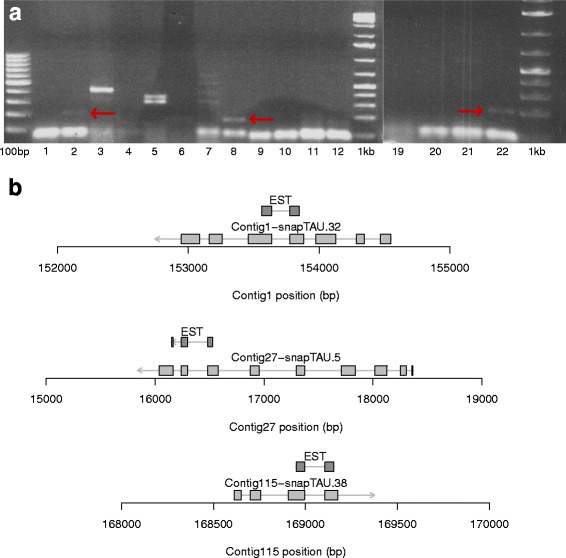
Validation of orphan genes. **a** PCR validation experiments for eleven candidate orphan genes. Genomic DNA (odd numbers) and cDNA (even numbers) was amplified using the same primer pairs. In three cases, we obtained bands in the expected size range. **b** Sequencing of amplification products resulted in ESTs that exactly confirmed the gene structure

Our previous analysis has indicated that a considerable number of orphan genes show evidence for negative selection. We combined our expression data with the selection analysis to screen for orphan genes that show negative selection but lack expression evidence (*N* = 550, FPKM = 0 in all 14 RNA-seq experiments). More precisely, the list of 3818 orphan genes showing significant negative selection were intersected with two other lists: first the list of 550 orphan genes without expression data and second the list of genes that had ω value less than 0.6 in at least 2 datasets (Fig. 3c). From the 29 genes found to be present in all three lists eleven genes that had more than 2 exons in their predicted open reading frame were selected as candidates for validation by PCR. PCR products from three candidate genes were found in the expected nucleotide range when PCR was done against cDNA but not against genomic DNA (Fig. 4a). Sequencing of PCR products resulted in expressed sequence tags that exactly matched the gene prediction (Fig. 4b). These results demonstrate, that even in the absence of expression evidence in 14 RNA-seq samples, PCR was able to detect expression of orphan genes in standard mixed-stage worm cultures. As expression profiles can be highly stage and tissue-specific, we speculate that further optimization of protocols and more specific RNA samples could validate predicted gene structures in many more cases.

### Differences between various gene classes

**Fig. 5:**
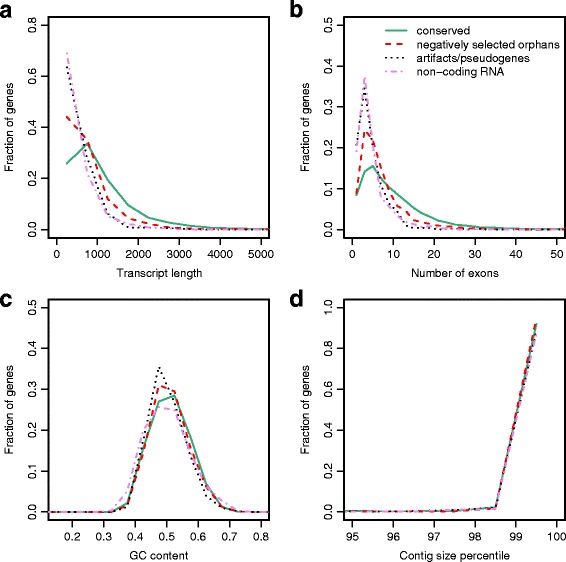
Differences between various gene classes. **a** Comparison of transcript length for potential prediction artifacts/pseudogenes, non-coding RNA candidates, negatively selected orphan genes, and conserved genes. The y-axis denotes the fraction of genes at a given transcript length. **b** Comparison of the number of exons for different gene classes. **c** GC content distribution for all four gene classes. **d** Distribution of contig size percentiles among all four classes. The top 1 % of largest contigs harbors roughly 90 % of genes for all four gene classes

Based on the obtained expression data and estimates for negative selection, we divided all *P. pacificus* genes into four classes. First class is made of 497 potential prediction artifacts or pseudogenes (orphans, without FPKM expression values above 1 and no sign of strong negative selection, i.e. ω < 0.6 in any of the analyses), second class contains 837 candidates for non-coding RNAs (FPKM > 10 in at least one RNA-seq data set, but no sign of strong negative selection), conserved genes and orphans that showed either significant negative selection or peptide evidence constitute the third and fourth classes. Four gene features (Transcript length, number of exons, GC content, and contig size percentile) were compared across all four gene classes (Fig. 5). While conserved and negatively selected orphans tend to be longer and contain more exons than potential prediction artifacts/pseudogenes and non-coding RNA candidates (*P* < 0.001, Wilcoxon test, Fig. 5a-b), at the level of GC content and contig size percentile no obvious differences are detectable (Fig. 5c-d). Given that fragmented assemblies were identified as potential source of prediction artifacts, it is interesting to see that in all four gene classes around 90 % of genes reside in the top 1 % of largest contigs. There are no significant trends towards accumulation of potential artifacts on smaller contigs. Thus, partial gene model as a result of smaller contigs are an unlikely source for the majority of prediction artifact or pseudogene candidates. However, further analysis is needed to characterize these gene sets in greater detail.

## Discussion

The drastic reduction in sequencing cost has facilitated whole genome sequencing of several non-model species. However, current genome sequencing projects constantly identify large fraction of genes without homology in related species. The amount of identified orphan genes depends on the definition as well as on the phylogenetic resolution of available genomic data. As a consequence, non-model or non-classical model organisms such as *P. pacificus,* which has been estimated to share a common ancestor with *C. elegans 280–430* MYA [23]*,* exhibit a much higher number of orphan genes in comparison to vertebrate genomes such as humans and mouse. Orphan genes can arise from technical (e.g. low resolution of phylogenetic) but also biological reasons (e.g. de novo gene formation). In addition, it has been shown that orphan genes can arise from genome assembly and gene prediction problems [18]. As gene prediction is still an integral part of current gene annotation pipelines [33], we need to first clarify whether orphan genes are truly protein-coding genes before we can study their biology and evolutionary history.

In this study, we use expression data to first test whether orphan genes are real biological entities or non-functional pseudogenes or gene prediction artifacts. First, we showed that up to 81 % of orphan genes are indeed transcribed. Second, using available proteomic and genomic data, we found that 40–77 % of orphan genes are actually truly protein-coding as they either show direct support in proteomic data or evolve under constraints that are expected for protein-coding genes. Although problems in gene annotation (e.g. fragmentation) still persist that might eventually be overcome by improvements in genome assembly and annotation, our study indicates that this will not eliminate the presence of orphan genes.

As the most prominent conclusion of our study, that orphan genes are protein-coding genes under negative selection is based on phylogenetic analysis, we would like to point out that most technical artifacts such as mispredictions, pseudogenes or incorrect alignment would drive the signal towards neutral selection. This suggests that further correcting these types of errors will increase the amount of detected evidence for negative selection. Furthermore, the finding of evolutionary constraint acting on orphan genes is consistent with other studies from insects, fish and primates [34–39]. Along with the accumulating functional evidence suggesting that taxonomically restricted genes can influence various phenotypic traits [14], recognizing orphan genes as true biological entities will further emphasize their importance and thus lead to a more holistic perspective in modern biomedical research.

Given that most orphan genes are protein-coding, the next questions we need to ask are: how do they evolve, how old are they, and what distinguishes conserved and orphan genes. Three possible scenarios for the emergence of orphan genes are frequently discussed in literature [6, 16]: strong divergence, horizontal gene transfer, and *de novo* gene formation. In *P. pacificus,* horizontal gene transfer has already been extensively studied [23, 40, 41] revealing strong evidence only for three different gene families. Thus, horizontal gene transfer is an unlikely explanation for the bulk of orphan genes in *P. pacificus*. It is difficult to decide on the remaining two scenarios because a clear proof of one scenario was only obtained by detailed case studies of individual genes [6, 42] and not by genome-wide analysis. Based on our analysis, we can only state that even if a period of strong divergence has lead to the loss of detectable homology, the evolutionary pressure that initially caused this divergence is not acting anymore at the moment since most orphan genes show evidence of strong negative selection.

As orphan genes have only been generally defined as taxonomically restricted genes [6], each individual study has to somewhat arbitrarily decide on a practical definition of orphan genes and the obtained results may depend on this definition. Here, we make use of previously identified orphan genes [22, 25], which were defined using 14 non-diplogastrid genomes as outgroup species. As the family of diplogastridae is comprised of several dozens of genera and is at least 20 million years old [43], the age of the identified orphan genes is probably quite variable and not comparable to other studies that defined orphans at a species-specific level [15, 35, 37]. To further investigate the age of *P. pacificus* orphan genes, future studies will have to incorporate genomic data at a much higher phylogenetic resolution. Our study also supports the hypothesis that the age of genes might be linked to the probability of a gene being embedded in gene-regulatory networks. A recent study in human and mouse [44] suggested that lineage-specific genes are initially loosely connected to the network periphery but with time become highly connected by acquiring essential and pleiotropic functions. It can be stipulated that the failure of an orphan gene to become an integral part of gene networks increases the possibility of its loss over long evolutionary periods. Based on our analysis we suggest that a substantial fraction of orphan genes exhibiting differential expression under various conditions are well integrated into gene networks and might be therefore much older than other recently evolved orphan genes that may have a transient functional role but eventually will be lost from the genome. Such a mechanism augurs well with the fact that even though new orphan genes are added to the genome still the genome size does not show proportionate increase.

Contrasting conserved genes with orphans, we find that orphan genes are shorter, have fewer exons, are less likely to be expressed, and are under less evolutionary constraint. Again, these findings are consistent with other studies from nematodes, insects, fish and plants [15, 34, 35, 37, 38, 45–50]. Although some of these features maybe related to the age of orphan genes, still further analysis is needed to investigate these trends and possible explanations in greater detail. Taken together, studies that prove the validity of orphan genes in individual genomes set up the starting point for further research of the biology and evolutionary history of orphan genes.

## Conclusions

Our study investigates the nature of orphan genes, which lack homology in other evolutionary lineages. In the case of the nematode *P. pacificu*s, we show that most of the predicted orphan genes are transcribed and evolve under constraints that are typical for protein-coding genes. Comparing paralogous and orthologous clusters with population genomic data suggests that the different data sets are highly complementary and demonstrates that even though evolutionary constraint acting on orphan genes can be studied within a single genome, the presence of a closely related genome has the greatest power for phylogenetic analysis. Our approach can be transferred to other genome data of non-model organisms in order to ascertain the biological importance of orphan genes.

## Methods

### Definition of orphan genes and homology clustering

Orphan genes were defined previously using BLASTP comparisons against 14 non-diplogastrid nematode outgroup genomes [25]. In summary, this procedure identifies orphan genes that are restricted to the family Diplogastridae and resulted in a total of 9885 orphan genes and 20999 conserved genes. We used the OrthoMCl software [31], with default parameters, to create homologous protein clusters between *P. pacificus* (version TAU) and *P. exspectatus* (version SNAP2012) genes. This allowed us to study selection at an interspecies (orthologous clusters) as well as intra-species level (clusters with paralogs). The clusters were divided into orphan and conserved gene clusters. We identified 253 cluster including both orphan and conserved genes of *P. pacificus*. Closer analysis of these hybrid clusters showed that events such as gene fusion, gene split, pseudogenisation and differential gene divergence rates can lead to mixing of orphan and conserved genes in a single cluster. We therefore decided to exclude these Hybrid clusters from further analysis.

### Analysis of transcribed and translated sequences

Expression of the predicted genes was assessed using previously generated RNA-seq [25] and micro-array data [28]. The dependence between the amount of genes with expression evidence and a number of analyzed RNA-seq samples was assessed using random permuations of RNA-seq samples (*N* = 14, Fig. 1a) and differentially expressed gene sets (*N* = 6, Fig. 1b).

Mass spectrometry data from earlier experiments [8, 30] was used to gather evidence for translation. Peptide data was compared with the predicted protein database using BLASTP. A predicted gene was considered as a translated gene, only if it had a BLASTP hit matching entire peptide length with 100 % identity.

### Divergence data between different *P. pacificus* lineages

In order to study selection at an even closer time-scale we used whole genome resequencing data for two deeply sampled *P. pacificus* clades [21], clade A1 from Asia (*N* = 15 strains), clade A2 from Southern and Central America and the Indian ocean (*N* = 16). These two clades are geographically isolated and show approximately 1 % genome-wide divergence from each other. We extract fixed differences to the reference genome (*N* = 485,795 for clade A1 and *N* = 618,650 for clade A2), which were covered in all sequenced strains per clade (by at least two reads with a samtools genotype quality above 20) and implanted them into the reference genome.

### Estimation of selection pressure

The predicted proteins from each cluster were aligned using MUSCLE [51] and the protein alignments were converted into codon alignments using pal2nal [52]. A minimum cutoff of 150 nucleotides length codon alignment was set to avoid bias introduced in the analysis by poor alignment or short peptides, as a result 231 clusters were removed from the analysis. RAXML was used to create the phylogenetic tree foe each cluster containing three or more proteins, for clusters smaller in size an unrooted tree was created. The Codeml package of the PAML suite [32] was run to first generate a single omega (ω) value for the entire tree (alternate model, H_A_) and then with a fixed ω value of 1 for the entire tree (Null model, H_0_).

In order to test the statistical significance of the estimated ω value, we performed Likelihood ratio tests (LRT) for each cluster. Considering that our null model is the model where ω is fixed at 1and our alternate model is where a single ω value is freely estimated for the entire cluster tree, the required assumption for a LRT i.e. that two models are nested is readily fulfilled. The test statistic is double the difference in log-likelihood (lnL) scores for the two models (LRT statistic = 2 (lnL H_A_ - lnL H_0_)). For large smaples, LRT statistic follows a chi-square distribution with degrees of freedom that is the difference in the number of freely estimated parameters between the alternate and null models. Here the only parameter that differs between the models is ω and thus the degree of freedom is 1. Hence the *p*-value of the LRT statistic was calculated from a chi-square distribution with a degree of freedom of 1 and then adjusted for false discovery rate (FDR). The alternate model was considered significantly better than the null model if the LRT statistic *p*-value (FDR adjusted) was less than 0.05.

The plots were generated using R [53] and Venn diagrams were generated using InteractiveVenn [54].

### Worm collection

*P. pacificus* worms were grown on nematode growth media agar plate and the *E. coli* strain OP50 was used as food source [55]. Adult worms were washed from the plate using M9 buffer and frozen at −80 °C with Trizol for RNA preparation and without Trizol for DNA extraction.

### DNA extraction

DNA extraction was performed using Sigma Gene Elute mammalin genomic DNA miniprep kit (Catalog number G1N70-1KT) as per manufacturer’s instructions.

### RNA preparation

Worm pellet frozen with Trizol was put through three freeze/thaw cycles in liquid nitrogen, followed by vigorous vortexing and 10 min incubation at room temperature. After centrifugation at 14000 rpm for 10 min at 4 °C, 100 μl chloroform was mixed with the supernatant, vortexed and incubated for 5 min at room temperature. The samples were again centrifuged at 14000 rpm for 15 min at 4 °C and the upper phase containing the RNA was transferred into a new tube. RNA precipitation was carried out by adding 0.5 μl glycogen blue and 250 μl isopropanol and then incubating at −20 °C for a few hours. The precipitated RNA was centrifuged at 14000 rpm for 10 min at 4 °C and then the pellet was washed with EtOH. Genomic DNA was digested using DNAse (RQ1). The dried pelleted RNAwas resuspended in 20 μl TE-buffer and incubated at 60 °C for 10 min to dissolve [27].

### cDNA synthesis

Invitrogen SuperScript® II Reverse Transcriptase kit (catalog number: 18064–014) was used to reverse transcribe cDNA as per manufacturer’s instructions.

### Candidate validation

PCR primers pairs ranging between 24 to 26 nucleotides in length were designed for all the candidates (Table 1) and then ordered to Eurofins for synthesis. PCR reaction was carried out using these primers against both *P. pacificus* genomic DNA and cDNA. The PCR program was 5 min at 94 °C, then 35 cycles of 3 steps - 94 °C for 30 s, 60 °C for 30 s and 72 °C for 30 s and then 72 °C for 10 min. The PCR products were run on 1.5 % agarose gel for 60 mins to check their size rang. Finally, the PCR products were sequenced using BigDye v3.1 Cycle Sequencing Kit (Catalog number: 4337457) from the Thermo Fischer Scientific as per instruction. The sequences derived from sequencing reaction were manually aligned to corresponding candidate regions.

**Table 1:** Primer pairs for the candidate genes

Gene identifier	Primer identifier	Primer sequence
Contig1-snapTAU.32	C1.32F1	TCTGTCCAGAGGAACGAATGGGATC
	C1.32R1	TGCACACTAACAAGTCTTCCCTCAG
	C1.32F2	CAGGAAAGATCGTCAAACAGGACCA
	C1.32R2	TGATTTCTCTTCAGGAGACACTCAG
Contig115-snapTAU.38	C115.38F1	GTCAGAGTGGAAATCAGTGCAACTG
	C115.38R1	TCACTTCCGTGTGTACGATTGACTT
	C115.38F2	ATGCCGAGCACAGAACAAATGCTGC
	C115.38R2	ACCGAGATTGCGGAAAACAGCGCAA
Contig159-snapTAU.23	C159.23F1	TTCATCGCTGACGATCACAGGCACA
	C159.23R1	AGATCATCATGCAGCCCTCCTTTGC
	C159.23F2	ATGCTCAAACTCCTCGTCTTCACCA
	C159.23R2	ACGATTTGACTGCGGGCTCTGCCTT
Contig162-snapTAU.8	C162.8F1	ATCAATGGCAATAAATCCGCTTACG
	C162.8R1	ATAAAGCCGTGAAGGTAATTCTCAT
	C162.8F2	AATAAATCCGCTTACGAACCAATCG
	C162.8R2	GGTAATTCTCATATTTGATGATTCC
Contig163-snapTAU.25	C163.25F1	GCAATCCCTCTACTGGCAGAATCTC
	C163.25R1	ATTGCATGGAGAGTACGTATCCGAC
	C163.25F2	AACTATGAAGGCGGTGATTCATTGG
	C163.25R2	GTTCGTTGAAAATCCACACTTTTCG
Contig27-snapTAU.5	C27.5F1	ACAAGAAGGCATACATGATGTACCC
	C27.5R1	AGTAGTCGAGGTGATGCTGTCAGGA
	C27.5F2	AACTGCATCTCAGACGCATCGGACA
	C27.5R2	TTTGACCTTGAACGCTTTCCTCCCG
Contig51-snapTAU.126	C51.126F1	ATGCTTGCGTGCATTGGGATCATCG
	C51.126R1	TAGCTCATTGAGATCAATGTCTTCG
	C51.126F2	TGACCTTCCTCGGCGGATGTTCCA
	C51.126R2	AGTTCACTTAGGCTCTCAAATGAGG
Contig57-snapTAU.76	C57.76F1	AGGAGATGATCGATAAACACAAAGCC
	C57.76R1	TCTTCTTCTGCAGCTGATTTGCCAC
	C57.76F2	TCGACAAGTGCTTCAAAGCCGAGCT
	C57.76R2	AAGATCCTCAAACTTCTCGCTGTG
Contig62-snapTAU.17	C62.76F1	TGCAAGTTGCACATCTCAACCACCT
	C62.76R1	ACACTTGGTTTCTTGAATGAGCTAAC
	C62.76F2	TGGGGATATCAAGTGCAAAGGCACTG
	C62.76R2	TTGGCTGGTTGGCTCTCGAATACTG
Contig67-snapTAU.30	C67.30F1	ATTCGACGTCTACTCTCACGCAACA
	C67.30R1	ATACGAAGTACAACATCACCTTGAG
	C67.30F2	TTCCGGCACACTTCTCATCATTCTC
	C67.30R2	AAATGAACGAGTACAACAGTAAACC
Contig68-snapTAU.138	C68.138F1	ACTGATTGCTGCTCATACAGATCGA
	C68.138R1	ACTGAGGAGCATCGTAAGCTGACTC
	C68.138F2	TCTTATTGGCTATACTGATTGCTGC
	C68.138R2	ATCCACTTTCCTGTCGAATTGACGC

## Abbreviations

FDR, False discovery rate; FPKM, fragments per kilobase transcript per milliion fragments sequenced; LRT, log-likelihood ratio test; ω = dN/dS, omega.
